# California ACEP Firearm Injury Prevention Policy

**DOI:** 10.5811/westjem.2020.11.50900

**Published:** 2021-01-20

**Authors:** Jorge Fernandez, Taylor Nichols, Zahir Basrai, Randall Young, Michael Gertz, Marc Futernick, Andrew Fenton

**Affiliations:** University of California, San Diego, Department of Emergency Medicine, San Diego, California

## Abstract

Firearm-related deaths and injuries are a serious public health problem in California and the United States. The rate of firearm-related deaths is many times higher in the US than other democratic, industrialized nations, yet many of the deaths and injuries are preventable. The California American College of Emergency Physicians Firearm Injury Prevention Policy was approved and adopted in 2013 as an evidence-based, apolitical statement to promote harm reduction. It recognizes and frames firearm injuries as a public health epidemic requiring allocation of robust resources, including increased governmental funding of high-quality research and the development of a national database system. The policy further calls for relevant legislation to be informed by best evidence and expert consensus, and advocates for legislation regarding the following: mandatory universal background checks; mandatory reporting of firearm loss/theft; restrictions against law-enforcement or military-style assault weapons and high capacity magazines; child-protective safety and storage systems; and prohibitions for high-risk individuals. It also strongly defends the right of physicians to screen and counsel patients about firearm-related risk factors and safety. Based upon best-available evidenced, the policy was recently updated to include extreme risk protection orders, which are also known as gun violence restraining orders.

Firearm-related injuries and deaths are a serious public health problem in the United States (US), yet the idea of regulating firearm ownership and access is complicated, politically charged, and potentially conflicts with US Constitution 2nd Amendment rights. The rate of firearm-related deaths is many times higher in the US than in other democratic, industrialized nations.^1^ In 2015, there were 113 firearm deaths per million individuals in the US as compared with 0.8 in the United Kingdom.^1^,^2^

Despite this disparity, and largely due to politics, firearm violence prevention research receives significantly less US federal funding compared with other leading causes of death; yet available research suggests that many firearm-related injuries and deaths are preventable.^3^,^4^,^5^,^6^ A 1993 study published in the *New England Journal of Medicine* and funded by the US Centers for Disease Control and Prevention (CDC) identified an association between elevated homicide risk within homes with guns. In response, the National Rifle Association (NRA) successfully lobbied US Congress in 1996 to include the “Dickey Amendment” in the federal omnibus spending bill.^7^ That amendment stripped $2.6 million from the CDC’s budget (the amount it had spent on firearm research the previous year) and added the following language: “none of the funds made available for injury prevention may be used to advocate or promote gun control.” Thereafter, federal firearm safety and violence research funding at the CDC, and later the National Institutes of Health (NIH), was effectively eliminated.^8^ A 2013 report from the Institute of Medicine concluded, “the scarcity of research on firearm-related violence limits policymakers’ ability to propose evidence-based policies that reduce injuries and deaths and maximize safety.”^9^ Using a methodology that calculated expected levels of research investment based on mortality rates, one study estimated that between 2004 and 2015 firearm violence prevention research received just 1.6% of the federal research support projected, and had just 4.5% of the volume of publications anticipated.^10^ Congress in 2018 clarified that the CDC can conduct research into firearm injury prevention, but again cannot use government funds to specifically advocate for gun control. Subsequently, the 2020 federal omnibus spending bill specifically allocated $25 million to the CDC and NIH toward firearm violence prevention research.^11^

Founded in 1971, the California Chapter of the American College of Emergency Physicians (California ACEP) is a 501(c)(6) non-profit, non-partisan, association representing California’s board-certified emergency physicians (EP). California ACEP’s mission is to support EPs in providing the highest quality of care to all patients and to their communities. In 2000, the California ACEP board of directors (BOD) voted to make firearms injury prevention one of the organization’s legislative priorities and approved a position statement concerning firearm injury prevention. In 2013, multiple bills regarding mandatory firearm restrictions were proposed to the California State Senate and Assembly. The California ACEP BOD tasked a subcommittee with reviewing the chapter’s position statement and available research, updating the chapter’s official policy, and guiding its legislative and advocacy efforts. The California ACEP Firearm Injury Prevention Policy (Firearm Policy) was approved and adopted in 2013 as an evidence-based, apolitical statement to promote harm reduction. The Firearm Policy recognizes and frames firearm injuries as a public health epidemic requiring allocation of robust resources, including increased government funding of high-quality research and the development of a national database system of firearm injuries. The policy further calls for legislation to be informed by best evidence and expert consensus, and advocates for legislation focused on the following:

1. Mandatory universal background checks
2. Mandatory reporting of firearm loss/theft
3. Restrictions against law-enforcement or military-style assault weapons and high capacity magazines
4. Child-protective safety and storage systems
5. Prohibitions against gun possession or purchase for high-risk individuals
6. The right of physicians to screen and counsel patients about firearm-related risk factors and safety.

In a subsequent review of the scientific literature on the effects of firearm injury prevention policies, the RAND Corporation cited evidence supporting child-access prevention laws, mandatory waiting periods, universal background checks, prohibitions related to domestic violence and mental illness, along with minimum age and licensing/permitting requirements.^6^ Notably, all these recommendations are included in the Firearm Policy.

In 2016, in response to recent highly publicized mass shootings including San Bernardino and Sandy Hook, the state of California overwhelmingly passed Proposition 63 (63% in favor vs 37% opposed).^12^ Proposition 63 focused mainly on the regulation of ammunition. It mandated a universal background check and California Department of Justice authorization to purchase ammunition (in addition to firearms, which was already regulated), and it specifically prohibited *possession* of large capacity magazines (LCM), which hold more than 10 rounds of ammunition. Prior to Proposition 63, it had been illegal in California to manufacture, purchase, receive, import, keep, sell, give, or lend LCMs. Proposition 63 also levied fines against firearm owners who fail to report the theft or loss of their firearm.^13^ Several regulations in Proposition 63, including a ban on LCM possession and mandatory reporting of firearm loss or theft, were advocated by the Firearm Policy. The NRA subsequently sponsored a legal challenge to Proposition 63 (DUNCAN v BECERRA),^14^ and in March 2019, the District Court for the Southern District of California ruled that Proposition 63 was unconstitutional, despite testimony by EPs on behalf of California ACEP. On August 14, 2020, a divided three-judge panel of the Ninth District Federal Court of Appeals upheld the federal district court’s ruling. That decision is currently being further appealed,^15^ and the case is being closely tracked by California ACEP’s BOD and staff.

Another crucial firearm-related violence prevention policy topic recently reviewed by the California ACEP BOD concerns extreme risk protection orders (ERPO), which are also known as gun violence restraining orders. In many states including California, medical professionals, law enforcement officers, coworkers, teachers, and family members may petition a court for ERPOs, which preemptively and temporarily authorize law enforcement officers to remove firearms from individuals deemed high risk for self-harm or violence against others. ERPO laws often allow formal court appeal and forbid harassment, to prevent misuse of ERPOs that could restrict access to firearms for defense, hunting, or recreation.^16^ Several studies examining ERPOs in states outside of California suggest that they are modestly effective in reducing firearm-related suicides.^17^ Per a RAND analysis, there were limitations in these studies, including the extrapolation of suicide attempts, rather than observed data, and a lack of comparison groups.^6^ However, the data was convincing enough to move the chapter’s BOD in 2020 to include ERPOs in an update to the Firearm Policy.

California ACEP strongly believes that it should advocate for evidence-based solutions to public health and policy issues, including firearm violence prevention and safety. Clearly, preventing injuries and deaths is more effective than, and preferable to, heroic saves in the emergency department or trauma bay. The Firearm Policy promotes evidence-based legislative recommendations and highlights the urgent need for more robust government funding, data, and evidence to effectively address the firearm violence epidemic in California and the US.

## California ACEP Firearm Injury Prevention Policy

It is the position of the California Chapter of the American College of Emergency Physicians that:

1. Emergency Medicine is well positioned, as a profession and specialty, to appreciate the multifaceted ramifications of firearm injuries in our society. Firearm violence is a public health epidemic that can only be effectively cured by deploying necessary and appropriate resources.
2. California ACEP deplores attempts to politicize or silence physicians and science on firearm violence. We recommend robust funding (federal and otherwise) of research on firearm injury and evidence-based prevention as well as its impact on public health and safety. It is our hope and belief that such research will guide better future legislation and lead to well-informed public policy.
3. Legislative measures and policies to curb or reduce firearm violence should be informed by evidence-based consensus. We advocate for continued research and implementation of programs focused on the safe storage of legitimate firearms, development of childproof or personalized guns, prevention of both interpersonal and self-directed violence by firearms, including the prevention of gang-related and domestic violence.
4. We support mandatory, comprehensive, and universal background checks for the purchase of firearms. Background checks should be required for essentially all firearm transfers, including at gun shows and auctions and from private sellers. Prohibited straw purchases of firearms should be recognized as serious crimes and be treated as such, and all secondhand gun sales and firearm transfers should be regulated. We support continued efforts to improve the quality of the data on which background checks are performed, such that all prohibited persons can be detected.
5. We support requiring that all firearm owners of record be required to report the theft or loss of their firearm within a timely period of becoming aware of such a loss.
6. We recommend legislation banning civilian purchase or access to assault weapons, large-capacity ammunition magazines, and any munitions specifically designed for the use by military and law enforcement agencies.
7. We encourage all healthcare providers, including emergency physicians, to screen and counsel patients with diagnosed mental illnesses or believed to be at risk of harming themselves or others for their potential access to firearms, and to refer such patients to appropriate mental health services in a timely manner. Policies and procedures for this process need to be validated and standardized.
8. We recommend the creation of a national database and surveillance system to track firearm-related injury and mortality, including mandatory reporting of firearm injuries and fatalities by all hospitals and healthcare centers.
9. We support restraining orders that allow for the removal of a firearm to provide a rapid, focused response when risk for imminent firearm violence, including suicide and homicide, is high. We support restraining orders that rely on actions by judicial officers and include due process protections and provide for immediate firearm recovery and include a prohibition on possession and purchase of firearms and ammunition. We support allowing petitions for such orders to be submitted by family members, law enforcement officers, physicians, and other mental health professionals including school counselors.
10. We recommend prohibiting firearm purchases by individuals in high-risk categories that include but are not limited to habitual criminals, drug traffickers, persons with mental illness who are suicidal or high risk, those with violent misdemeanors, persons with multiple convictions for alcohol-related offenses, those with a history of domestic violence, juveniles convicted of violent crimes, and violators of parole and restraining orders.
11. We believe in the protection of healthcare providers’ rights to educate patients regarding firearm safety. We encourage all healthcare providers, including emergency physicians, to counsel patients about firearm safety when appropriate including discussing with parents safe storage of firearms in homes with children.
